# Self-Care for Common Colds: The Pivotal Role of Vitamin D, Vitamin C, Zinc, and* Echinacea* in Three Main Immune Interactive Clusters (Physical Barriers, Innate and Adaptive Immunity) Involved during an Episode of Common Colds—Practical Advice on Dosages and on the Time to Take These Nutrients/Botanicals in order to Prevent or Treat Common Colds

**DOI:** 10.1155/2018/5813095

**Published:** 2018-04-29

**Authors:** Mariangela Rondanelli, Alessandra Miccono, Silvia Lamburghini, Ilaria Avanzato, Antonella Riva, Pietro Allegrini, Milena Anna Faliva, Gabriella Peroni, Mara Nichetti, Simone Perna

**Affiliations:** ^1^Department of Applied Health Sciences, Azienda di Servizi alla Persona (ASP) di Pavia, University of Pavia, Pavia, Italy; ^2^Research and Development Unit, Indena, Milan, Italy

## Abstract

Maintaining a normal healthy immune defense system lowers the incidence and/or the severity of symptoms and/or the duration of common cold (CC). Physical barriers and innate and adaptive immunity have been involved during a CC episode. Vitamins C and D, zinc, and* Echinacea* have evidence-based efficacy on these immune system barriers. This review includes 82 eligible studies to consider the preventive role of these nutrients in immune clusters and in CC to provide advice on dosage and assumption of these nutrients. Regarding vitamin C, regular supplementation (1 to 2 g/day) has shown that vitamin C reduces the duration (in adults by 8%, in children by 14%) and the severity of CC. Considering zinc, the supplementation may shorten the duration of colds by approximately 33%. CC patients may be instructed to try zinc within 24 hours of onset of symptoms. As for vitamin D, the supplementation protected against CC overall, considering baseline levels and age. Patients with vitamin D deficiency and those not receiving bolus doses experienced the most benefit. Regarding* Echinacea*, prophylactic treatment with this extract (2400 mg/day) over 4 months appeared to be beneficial for preventing/treating CC. In conclusion, the current evidence of efficacy for zinc, vitamins D and C, and* Echinacea* is so interesting that CC patients may be encouraged to try them for preventing/treating their colds, although further studies are needed on this topic.

## 1. Introduction

Common cold (CC) is a conventional term used for mild upper respiratory illnesses, which comprises a heterogeneous group of self-limited diseases caused by numerous viruses and is the most frequently encountered human diseases worldwide [1].

In European populations, adults have from 2 to 5 infections annually, children typically present 6 to 12 “colds” per year, and rates of symptomatic infections increase in the elderly [2]. A seasonal variation is present with more episodes in winter and fall [1] and, on average, episodes of common colds last around 10 days [3].

Beyond impairing the quality of life [4], common colds have a tremendous economic burden on societies due to work absenteeism [5, 6].

Thus, treatments that reduce the incidence of infection and/or lessen the severity of symptoms and/or shorten the duration of common colds are of high interest both for the individual and for the whole society.

Maintaining the immune defense system within a normal healthy state lowers the incidence of infection and/or lessens the severity of symptoms and/or shortens the duration of common colds. Natural killer cell (NK cell) activity and salivary immunoglobulin A (IgA) are considered important in the prevention of common colds [7]. However, several environmental factors, including a stressful lifestyle, are likely to weaken the immune defense system [8], which may result in an increased risk of common cold. Lifestyles and mental health status are associated with natural killer cell and lymphokine-activated killer cell activities [8]. The immune system is an intricate network of specialized tissues, organs, cells, and chemicals protecting the host from infectious agents and other noxious insults. Although these defense mechanisms are very complex, they can be described as being organized in three main interactive clusters: physical barriers, and innate and adaptive immunity [9, 10].

The first barrier against “invaders” consists of physical barriers (low pH caused by various fatty acids and enzymes; it can limit the growth of most bacteria), mucus secretion (it contains proteins that can destroy pathogens), and the acidity of the stomach. Innate immunity is the second barrier and includes immune system cells, such as NK cells, cytokines (such as interferon-*γ*), macrophages, and neutrophil granulocytes. In addition, zinc shows its antiviral effects through the Intercellular Adhesion Molecule 1 (ICAM-1). Adaptive immunity is the third barrier to infection and is acquired later in life, such as after an immunization or successfully fighting off an infection. It retains a memory of all the invaders it has faced and this accelerates antibody production [11]. It includes lymphocytes T (e.g., regulatory T cells) and lymphocytes B.

### 1.1. Mechanism of Innate Immunity (Physical Barriers and Immune Cells) during Common Cold

When a respiratory virus is inhaled it first binds to nonspecific receptors on the respiratory epithelium, usually glycolipids or glycoproteins. Membrane fusion or endocytosis follows, thus internalizing the virus and enabling subsequent replication, transcription, and translation of new viruses which can then be released to infect new cells. Once a cell has been infected, pathogen-associated molecular patterns (PAMPs) on the virus can be recognized by various intracellular innate pathogen recognition receptors (PRRs) such as the toll-like receptors (TLRs), retinoic-acid-inducible gene-I- (RIG-I-) like receptors (RLRs), and nucleotide binding-oligomerization domain (NOD-) like receptors (NLRs) [12]. Pulmonary epithelial cells have been shown to express all of the known human TLRs and RLRs that detect viruses, and ligands for these PRRs activate epithelial cells in order to initiate a rapid immune response against viral invasion [13]. In addition to direct infection of epithelial cells, intraepithelial dendritic cells (DCs) residing just below the respiratory epithelium and tissue-resident macrophages continually sample particles in the airway lumen and can internalize them by phagocytosis and macropinocytosis, thus activating PRRs and initiating an immune response [14, 15].

Features of the induced antiviral state include resistance to viral replication in all cells, induction of apoptotic cell death in infected cells, increased major histocompatibility complex (MHC) class I expression to enhance antigen presentation, activation of dendritic cells (DCs) and macrophages, and stimulation of natural killer (NK) cells to enhance their cytolytic activity [16]. The inflammatory cytokines TNF-*α*, IL-1*β*, IL-6, and IL-12 are also produced at an early stage of the innate immune response. These cytokines promote leukocyte extravasation by increasing endothelial expression of adhesion molecules increasing vascular permeability, induce synthesis of acute phase proteins, and contribute to recruitment and activation of cells of the adaptive immune response [12]. Other antimicrobial vitamin D dependent peptides, such as cathelicidins and defensins, are involved in the second barrier.

### 1.2. Mechanism of Adaptive Immunity (Antibodies) during Common Cold

Around 72 h after infection, DCs with antigen-MHC complexes migrate through the afferent lymph vessels to secondary lymph nodes where they form interactions with naive CD4 and CD8 T lymphocytes. These T lymphocytes activate, proliferate, and differentiate into effector T cells and migrate via efferent lymph vessels into the circulation. Multiple chemokines are expressed in the respiratory epithelium and result in changes in integrin affinity, allowing effector T cells to bind to the endothelium and migrate into the infected tissue [17–19]. For efficient and effective viral clearance Th1 effector T cells are required, which produce IL-2, TNF-*α*, and IFN-*γ* to activate NK cells and induce generation of cytolytic molecules. CD8 effector T cells and NK cells can then induce apoptosis of infected cells [17]. B cells have also been demonstrated to play an important role in the immune response to highly pathogenic viral infections. Contact between CD4 T cells and naive B cells in secondary lymphoid tissues results in their proliferation and antibody class-switching, with neutralizing virus-specific antibodies crucial for optimal viral clearance. Additionally, viral components expressed on infected cells allow antibodies to bind, thus initiating antibody-dependent cell-mediated cytotoxicity (ADCC), whereby CD16 on NK cells recognizes the Fc portion of antibodies bound to the surface and kills the target cell [12, 17, 20, 21].

### 1.3. Self-Care for Common Colds: Role of Nutrition and Botanicals and Their Interaction in Three Main Immune Interactive Clusters: Physical Barriers, Innate and Adaptive Immunity

As common colds have a self-limited course and resolve without treatment, studies on self-care have shown that common colds are the most frequent cause for self-care [22–24]. A variety of alternative and nonpharmacologic treatments of the common cold are proposed [25, 26]. Between these numerous nonpharmacological approaches for prevention and treatment of the common cold, there are the intakes of some nutrients, such as zinc, selenium, iron, copper, b-carotene, vitamins A, D, C, and E, folic acid, and botanicals, such as* Echinacea* [26]. The proposed biologic mechanisms are that these nutrients can significantly influence several components of immunity [10]. But among these numerous nutrients, which have proven to have evidence-based efficacy on all three immune system barriers? The nutrients are vitamin C, vitamin D, and zinc, because all three nutrients have specific EFSA (European Food Safety Authority) scientific opinion on the substantiation of health claims related to vitamin D [27], vitamin C [28], zinc [29], and normal function of the immune system. Moreover, there is EFSA scientific opinion on the substantiation of health claims related to zinc [30] and to vitamin C [31] and maintenance of normal physical barriers, the first immune system barriers. Finally, for vitamin C [32] and* Echinacea *[33] there are Cochrane reviews regarding the use of these two nutrients for preventing and treating the common cold.

Given this background, the purpose of this narrative review is to consider the pivotal role of vitamin D, vitamin C, zinc, and* Echinacea* on three main immune interactive clusters (physical barriers, innate and adaptive immunity) in terms of prevention and treatment (shortening the duration and/or lessening the severity of symptoms) of common colds in order to provide practice advice on the dosages and on the time to take these nutrients.

## 2. Methods

The present systematic review was performed following the steps by Egger et al. as follows [34]: (1) A working group was configured as follows: three operators skilled in endocrinology and clinical nutrition, of whom one acting as a methodological operator and two participating as clinical operators. (2) The revision question on the basis of considerations made in the abstract was formulated as follows: “the state of the art on role of vitamin D, vitamin C, zinc, and* Echinacea* in three main immune clusters (physical barriers, innate and adaptive immunity) in terms of prevention and treatment (shortening the duration and/or lessening the severity of symptoms) of common colds.” (3) Relevant studies were identified as follows: a research strategy was planned, on PubMed [Public Medline run by the National Center of Biotechnology Information (NCBI) of the National Library of Medicine of Bethesda (USA)], as follows: (a) definition of the key words (common cold,* Echinacea*, immunity, nutrients, vitamin C, vitamin D, and zinc), allowing the definition of the interest field of the documents to be searched, grouped in quotation marks (“…”), and used separately or in combination; (b) use of the Boolean AND operator that allows the establishment of logical relations among concepts; (c) research modalities: advanced search; (d) limits: time limits: papers published in the last 30 years; languages: English; (e) manual search performed by the senior researchers experienced in clinical nutrition through revision of reviews and individual articles on role of vitamin D, vitamin C, zinc, and* Echinacea* in three main immune interactive clusters (physical barriers, innate and adaptive immunity) in terms of prevention and treatment (shortening the duration and/or lessening the severity of symptoms) of common colds published in journals qualified in the Index Medicus. (4) The analysis was carried out in the form of a narrative review of the reports.

## 3. Results

### 3.1. Zinc and the Three Main Immune Interactive Clusters (Physical Barriers, Innate and Adaptive Immunity) Involved during an Episode of Common Colds

This research has been carried out based on the following keywords: “zinc” OR “zinc supplementation” AND “immune response” AND “innate immunity” AND “adaptive immunity” AND “respiratory tract infections” AND “common cold” AND “Immunodeficiency.”

**Figure 1:**
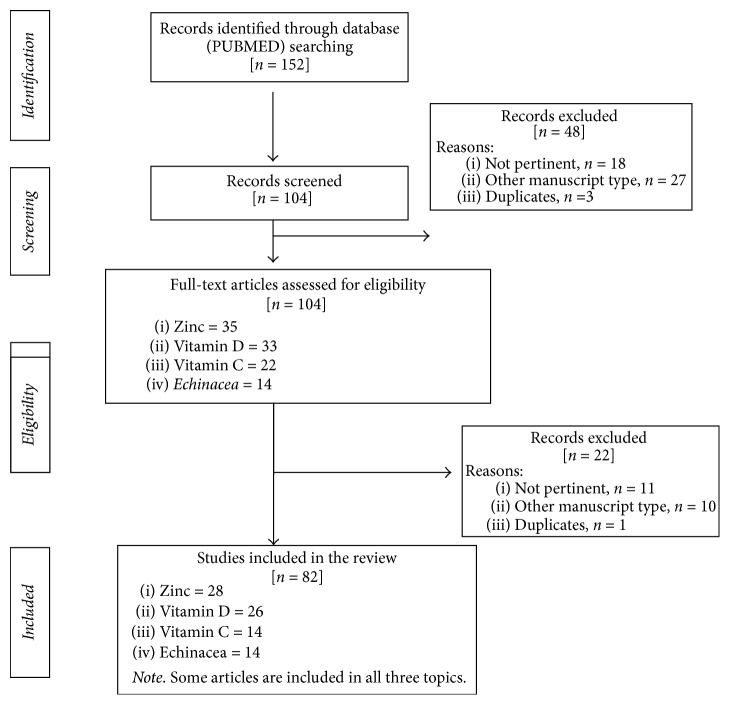
Flow chart of the study selection process.

Figure 1 shows the study selection process.

Table 1 summarizes the studies presented in the narrative review.

#### 3.1.1. First Barrier: Physical Barrier

In the present era, approximately two billion people in developing countries suffer from Zn deficiency (ZnD), mainly due to malnutrition and manifest clinical characteristics of growth retardation and compromised immune systems [35].

Adequate zinc intake helps to maintain physical barriers and mucosal membrane integrity and unbound zinc ions exert a direct antiviral effect on rhinovirus replication [10]. Supplementation of Zn improves immune functions, including delayed cutaneous hypersensitivity in children (10–20 mg of Zn) [36, 37], but other authors do not completely agree [38, 39]. It improves delayed-type hypersensitivity (DTH) in children supplemented with 10 mg/day [40].

#### 3.1.2. Second Barrier: Cellular Natural Immunity

Zinc supplementation increases cellular components of innate immunity (e.g., phagocytosis by macrophages and neutrophils, natural killer cell activity, and generation of oxidative burst) [10].

1. Neutrophil granulocytes, macrophages: large amounts of oral zinc significantly impaired polymorphonuclear leukocytes (PMNL) function and, in vitro, zinc potentiated the neutrophil response against* Staphylococcus aureus* [11]. Zn supplementation (150 mg/d) in elderly also induces a decrease in granulocyte zinc that has implications in phagocytosis and chemotaxis [41].
2. Natural killer: a supplementation of zinc (in vitro studies or 100 mg/d in elderly) improves natural killer (NK) cells activity, as argued by a lot of authors [9, 11, 39, 42]. Zinc administration decreased peripheral blood NK cell activity in vitro in patients with that inflammatory disease [11].
3. Cytokine: common cold viruses increase oxidative stress, which activates macrophages and monocytes and results in increased production of both the inflammatory cytokine IL-1*α* and the anti-inflammatory product IL-1ra [43]. After zinc restriction, there was reduction in the in vitro secretion of interleukin-2 receptor (IL-2R) [44]. Zinc is involved in the cytosolic defense against oxidative stress (superoxide dismutase activity) [10]. Recently, the transcriptional repressor Gfi1, a zinc finger protein, was identified as a regulator of immune response [45]. Inflammatory cytokines, such as tumor necrosis factor *α* and interleukin (IL) 1, are also known to generate greater amounts of reactive oxygen species and these parameters significantly decrease after zinc supplementation in the elderly (45 mg) [46]. Zn could normalize the production of interleukins, such as IL-2 [9], but also IL-1, IL-6, and tumor necrosis factor *α* (TNF-a), by mononuclear cells in vitro [11]. It modulates cytokine release by peripheral blood nuclear cells [26]. Il-6 increased in infants born to mothers that received Zn supplementation [47]. Most important, the ex vivo generation of TNF-*α* from isolated mononuclear cells (MNCs) is significantly decreased in elderly subjects after zinc supplementation [46]. Finally, Zn has role in the production of interferon-*γ* [48]. Zinc is involved also in the biosynthesis of leukotriene B4, which is implicated in a variety of acute and chronic inflammation, including chronic-obstructive pulmonary disease (COPD) [45]. The antiviral effects of zinc (5 mg), through the Intercellular Adhesion Molecule 1 (ICAM-1) receptor blocking, have been considered as one of the most important actions for impacting on incidence and/or duration of upper respiratory tract infections (URTI) [49].

#### 3.1.3. Third Barrier: Adaptive Immunity

Zn is required for proper antigen presentation via MHC-II to elicit adaptive immune responses [35]. Zinc deficiency in experimental animals is associated with low thymic weight and progressive loss of T lymphocytes (T cells) because zinc is an essential cofactor for the thymic hormone thymulin. Thymulin induces several T cell markers and promotes T cell function, including allogenic cytotoxicity, suppressor functions, and interleukin-2 production [26]. Zn supplementation (30 mg/day) is required in order to enhance functions of T cells, thanks to an increase in the production of T cells and/or a decrease in the loss of T cell precursors via apoptosis [50]. It increases the number of CD4 (helper) lymphocytes in children with a 10–20 mg of supplementation [36] or with 5 mg of zinc [51] and also CD8 [9, 26]. Since it is an essential cofactor for thymulin [10], a possible explanation of these effects on T cells is related to thymulin levels, which is required for the differentiation of CD4+ T cells [45, 52]. Another explanation is a direct effect of zinc ion on the lymphocyte membrane affecting maturation and differentiation of T lymphocytes [37]. Zinc acts on T lymphocytes through modulating IL-2 secretion, receptor expression, and sensitivity [45]. Zn controls antibody-mediated humoral immune responses, and a zinc cell-membrane-localized transporter (ZIP10-Zn) has a role in early B-cell development and the maintenance of mature B cells [35].

#### 3.1.4. Zinc Supplementation for Common Colds

Many studies have agreed that supplementation of zinc is helpful in reducing the risk of pneumonia and common cold and the incidence of respiratory tract infection, specifically in the elderly and in children [9, 33, 36, 50, 53, 54]. Zinc supplementation (20 mg/day) accelerates recovery from severe pneumonia in children [42]. (However, two articles were found which do not support a role for intranasal zinc (gluconate) in prevention or treatment of the common cold or immune parameters [55, 56], but in these two studies the way of administration is intranasal.) Very recently two meta-analyses demonstrated that zinc may shorten the duration of colds by approximately 33% [57]. However, there are some topics about zinc and common cold that are still not clear. In particular, high dosage of zinc in clinical trials has caused adverse effects, such as bad taste, and the variation in the total daily dose of zinc.

In conclusion, given the discreet evidence of efficacy on shortening the duration of colds by approximately 33%, common cold patients may be instructed to try zinc within 24 hours of onset of symptoms [57]. However, since controlled trials that have examined the effect of zinc on the common cold have diverged, the optimal composition and dosage of zinc should be better investigated in addition to the optimum frequency of their administration.

### 3.2. Vitamin D and Three Main Immune Interactive Clusters (Physical Barriers, Innate and Adaptive Immunity) Involved during an Episode of Common Colds

This research has been carried out based on the following keywords: “vitamin D” OR “vitamin D supplementation” AND “immune response” AND “innate immunity” AND “adaptive immunity” AND “respiratory tract infections” AND “common cold” AND “immunodeficiency.”

Figure 1 shows the study selection process.

Table 2 summarizes the studies presented in the narrative review.

#### 3.2.1. First Barrier: Physical Barrier

The active hormone 1,25(OH)2D is important in upregulating genes via the 1a-hydroxylase enzyme, which then encode proteins required for tight junctions (e.g., occludin), gap junctions, and adherens junctions (e.g., E-cadherin) [58].

Vitamin D supplementation increases cathelicidin production and it is involved in the production of defensins [59]. These antimicrobial peptides are also involved in the second barrier.

#### 3.2.2. Second Barrier: Cellular Natural Immunity

A review by Hewison et al. shows that vitamin D supplementation is successful in raising serum levels of 25OHD in TB patients and it may also play a role in promoting innate immune responses to enhance monocyte phagocytosis and degradation of b-amyloid.

The effects of vitamin D3 on macrophage phagocytosis may be related to the ability of that vitamin to alter monocyte maturation. Thus, D3 enhances immunoglobulin and complement-mediated phagocytosis by human monocytes through its stimulation of monocyte maturation to macrophages [11].

Enhancing protective innate immune responses, 1,25(OH)2D helps maintain self-tolerance by dampening overly zealous adaptive immune responses.

In addition, oral supplementation with HyD (25(OH)D3 metabolite) at a dose of 20 *μ*g per day may explain the benefit of HyD on systolic blood pressure reduction, improvement in lower extremity function, and the more pronounced reduction in several markers of innate immunity among healthy postmenopausal women [60].

#### 3.2.3. Third Barrier: Adaptive Immunity

Human epidemiological studies indicate supplementation with 1,25(OH)2D3 as an independent protective factor influencing the occurrence of Th-1 mediated autoimmunity [10]. The effects of 1,25(OH)2D on the immune system include decreasing Th1/Th17 CD4+ T cells and cytokines, increasing regulatory T cells, downregulation of T cell-driven IgG production, and inhibition of dendritic cell differentiation [61]. The adaptive immune effects of vitamin D are not restricted to effector T cells and also include actions on suppressor or regulatory T cells (Treg), a group of CD4+ T cells known for inhibiting the proliferation of other CD4+ T cells. Treatment of naive CD4+ T cells with 1,25(OH)2D potently induces the development of Treg, and this may exert beneficial effects in autoimmune disease and host-graft rejection [62].

A short term high-dose vitD3 supplementation (140.000 IU) significantly increased the frequency of regulatory T cells (Tregs) but did not further improve *β*-cell function in apparently healthy subjects.

VitD3 may be a useful therapeutic agent in autoimmune diseases exerting immune modulatory effects involving stimulatory actions on Tregs [63].

Vitamin D deficiency or insufficiency has immunological implications in patients with recurrent miscarriage (RM). The percentage of B cells, the percentage of TNF-*α*-producing Th cells, and NK cytotoxicity are significantly reduced under 0.5 *μ*g/day of 1,25(OH)2D supplementation for 2 months [64]. Treatment with 4000 IU vitamin D3 significantly reduced CD4+ T cell activation compared to low-dose vitamin D3, providing human evidence that vitamin D can influence cell-mediated immunity [65].

Vitamin D associated with upper respiratory tract infections (URI) burden probably involves lymphocytes and their activity. However, thymus activity, represented by higher T cell receptor excision circles (TREC, markers of thymus activity) levels, is not related to vitamin D concentrations or status and is not affected by 2000 IU/d vitamin D supplementation in adolescent swimmers [66].

#### 3.2.4. Vitamin D and Common Colds

Clinical trials demonstrate that 400 IU/d vitamin D supplementation is needed for the prevention of respiratory infections [67, 68]. Vitamin D supplementation decreases the events related to respiratory tract infections. In particular, vitamin D is useful in prevention of these types of infections, assuming dosage of vitamin D ranging from 400 IU/day to 2000 IU/day [68]. Epidemiologic studies have found high vitamin D levels to be associated with lower risk of infections of the upper respiratory tract (colds). 4000 IE/day vitamin D supplementation is found to significantly increase the probability of staying infection free during the study period. This finding further supports the notion that vitamin D status should be monitored in adult patients with frequent respiratory tract infections, and patients with vitamin D deficiency must be supplemented [69]. The more vitamin D is reserved within the infants' bodies, the more they will be immune to respiratory infections. It is assumed that the lack of significant differences in vitamin D is due to the gestational age and other factors except that vitamin D deficiency plays crucial roles in respiratory system infections [70]. Supplementation with vitamin D in children seems to be a strong ally in fighting the onset of respiratory infections. The combination of vaccines and vitamin D supplementation can significantly reduce the appearance of URTIs and the use of antibiotics, with a consequent decrease of global indicators of bacterial resistance [71]. In Mongolian children, who received milk fortified with 300 IU of vitamin D3, vitamin D supplementation significantly reduced the risk of acute respiratory infections (ARIs) in winter among children with vitamin D deficiency [72]. Finally, 1200 IU/d vitamin D3 supplementation during the winter may reduce the incidence of influenza A and enhance innate immunity by upregulating antimicrobial peptides, especially in specific subgroups of schoolchildren [73]. Weekly supplementation with 10,000 IU of vitamin D3 is preventive for URTI in young adults [74].

Vitamin D supplementation is safe and protected against acute respiratory tract infection overall, but patients who are very deficient in vitamin D and those not receiving bolus doses experienced the most benefit, as demonstrated very recently in a meta-analysis that considered 25 eligible randomized controlled trials (total 11,321 participants, aged 0 to 95 years) [75].

On the other hand, some evidence shows no significant correlation between vitamin D levels and lower respiratory tract infections in terms of the disease and its severity [76], despite a 50 *μ*g vitamin D3 (2000 IU) daily supplementation [77]. The sub-sunburn sunbed treatment is effective in tanning and increasing the 25(OH)D serum level, more so than 1000 IU per day, but had no appreciable effect on colds [78]. In patients with mild to moderate asthma undergoing an inhaled corticosteroid dose reduction, the use of vitamin D supplementation (100,000 IU load plus 4,000 IU/d) is not supported for the purpose of reducing cold severity or frequency [79]. In addition, monthly administration of 100,000 IU of vitamin D does not reduce the incidence or severity of URTIs in healthy adults [80]. It is reasonable and safe to take approximately 1000 IU of vitamin D daily, as suggested by Zittermann et al., in order to optimize nonspecific immunity and prevent infection. It is important to start supplementation in early autumn in order to ensure an adequate vitamin D level in winter [81].

In conclusion, vitamin D supplementation was safe and it may protect against acute respiratory tract infections overall, although there are numerous studies that do not support this indication and therefore it is necessary that further research will be conducted on the dosage of intake of vitamin D and prevention/treatment of common cold. Baseline levels of vitamin D, age, and dose of vitamin D need to be taken under consideration in order to personalize therapy. Patients who were very deficient in vitamin D and those not receiving bolus doses experienced the most benefit, and it is important to start supplementation in early autumn in order to ensure an adequate vitamin D level in winter.

### 3.3. Vitamin C and Three Main Immune Interactive Clusters (Physical Barriers, Innate and Adaptive Immunity) Involved during an Episode of Common Colds

This research has been carried out based on the following keywords: “vitamin D” OR “vitamin D supplementation” AND “immune response” AND “innate immunity” AND “adaptive immunity” AND “respiratory tract infections” AND “common cold” AND “immunodeficiency.”

Figure 1 shows the study selection process.

Table 3 summarizes the studies presented in the narrative review.

#### 3.3.1. First Barrier: Physical Barrier

1 study was focused only on the physical barriers [82] and the aim of this study was to measure changes in the radical-scavenging activity of human physical barriers in vivo due to supplementation with different doses of vitamin C (100 or 180 mg) and at different time points. The study shows that orally administered vitamin C can have a significant radical-scavenging effect on physical barriers.

#### 3.3.2. Second Barrier: Cellular Natural Immunity

5 studies observed an improvement in the innate immune function [83–87].

Specifically, Schertling et al., 1990 [84] used a high dose of ascorbic acid (5 g/die) and observed that additional administration of ascorbic acid and over a longer period of time may be expected to provide a therapeutic effect in the presence of increased activity of the pulmonary inflammatory cells (e.g., alveolar macrophages, granulocytes) with bronchial asthma. Harper et al., 2002, have selected, as an outcome, the reduction in spontaneous generation of superoxide and total antioxidant capacity observing reduced neutrophil generation of superoxide [85]. In the study of Du et al., 2003, two different doses (10 g/day or 1 g/day) have been used in patients with pancreatitis and have demonstrated therapeutic efficacy [86]. The potential mechanisms include promotion of antioxidizing ability of pancreatitis patients, blocking of lipid peroxidation in the plasma, and improvement in cellular immune function. Vojdani et al., 2000, using three different doses of ascorbic acid (500, 1000, or 5000 mg), did not observe adverse effects on the activity of NK cells even at high doses (5000 mg) [87]. In four studies an improvement in the innate immune function was not observed [88–91]. Nieman et al., 2002, used a dose of 1500 mg and focused on immune changes after an ultramarathon, showing that supplementation of vitamin C does not serve as a countermeasure to postoxidative race and immune changes in carbohydrate fed ultramarathon runners [88]. Davison and Gleeson [89] used a dose of 1000 mg, and the aim of study was to determine the effect of 2 weeks of supplementation with vitamin C on cortisol, adrenocorticotrophic hormone, interleukin-6, oxidative stress, and neutrophil responses to a single bout of endurance exercise. The authors concluded that supplementation with vitamin C for a maximum period of two weeks provides very limited or no protection against depression neutrophil function that is typically seen after prolonged exercise. The study of Hunter et al., 2012, [90] aimed to assess whether regular consumption of gold kiwifruit reduces upper respiratory tract infections (URTI) symptoms in older people and determined the effect it has on plasma antioxidants and markers of oxidative stress, inflammation, and immune function. The results showed that no changes to innate immune function (natural killer cell activity, phagocytosis) or inflammation markers (high-sensitivity C-reactive protein, homocysteine) were detected. The results of the pilot study of McComsey et al., 2003, conducted in HIV-infected subjects with lipoatrophy demonstrated that antioxidant supplementation did not change significantly immunity [91].

#### 3.3.3. Third Barrier: Adaptive Immunity

Only one study in allergic adults was focused on the acquired immunity [92] and used a dose of 1500 mg; the aim was to determine the effects of dietary antioxidants on allergen-specific immune responses in sensitized individuals. The study found that antioxidant supplementation resulted in significant increases in serum levels of vitamin C, vitamin E, *β*-carotene, and selenium levels, compared with the placebo group, but here there was no change in serum antioxidative capacity (AC), plasma F2-isoprostanes, exhaled nitric oxide (eNO), or immune responses following supplementation with antioxidants compared with placebo.

Penn et al., 1991, have used a dose of 100 mg by observing an improvement in the immune function cell-mediated immunity, in particular T lymphocyte [83].

#### 3.3.4. Vitamin C and Common Colds

The Cochrane review by Hemila et al., 2011, encompassing twenty-nine trials with 11,306 research participants, concluded that regular ingestion of vitamin C had no effect on common cold incidence in the ordinary population [32]. However, it had a modest but consistent effect in reducing the duration and severity of common cold symptoms: in adults the duration of colds was reduced by 8% (3% to 12%) and in children by 14% (7% to 21%); moreover, in children, 1 to 2 g/day vitamin C shortened colds by 18%. Maggini et al., 2012, used a dosage of 1000 mg plus 10 mg zinc and showed that supplementation with vitamin C and zinc may represent an efficacious measure, with a good safety profile, to help ameliorate the symptoms of this infectious viral disease [54].

A particular category of subjects that may need vitamin C supplements is athletes who engage in heavy physical activity, as these athletes' vitamin C status may be depleted. A review demonstrated that in trials with participants exposed to short periods of extreme physical stress (including marathon runners and skiers), supplementation with vitamin C (0.6–1.0 g/day) halved the common cold risk [93]; the results of this review suggest that vitamin C supplementation may be beneficial for some of the subjects doing heavy exercise who have problems with frequent upper respiratory infections. An important point to consider is that overdosing vitamin C might be negative for immune defense as it inhibits oxidative processes that are needed for first line defense against bacteria or viruses [94–96].

In conclusion, regular supplementation (1 to 2 g/day) has shown that vitamin C may reduce the duration (in adults by 8%, in children by 14%) and the severity of CC. Therefore, given the low cost and safety, it may be also worthwhile for common cold patients to test on an individual basis whether therapeutic vitamin C is beneficial for them, considering that under certain conditions vitamin C can act as a prooxidant, potentially contributing to oxidative damage.

### 3.4. *Echinacea* and Three Main Immune Interactive Clusters (Physical Barriers, Innate and Adaptive Immunity) Involved during an Episode of Common Colds

This research has been carried out based on the following keywords: “*Echinacea*” AND “immune response” OR “innate immunity” OR “adaptive immunity” OR “respiratory tract infections” OR “common cold” OR “immunodeficiency.”

#### 3.4.1. First Barrier: Physical Barrier

*Echinacea* extracts have the immunomodulatory potency to promote both phenotypic and functional maturation of murine dendritic cells via modulating the activation of JNK, p38-MAPK, and NF-*κ*B pathways [97]. A similar effect has been demonstrated in human dendritic cells [98].

#### 3.4.2. Second Barrier: Cellular Natural Immunity

*Echinacea* significantly normalized the restraint stress-induced reduction in splenocyte proliferation and splenic natural killer (NK) cell activity in rats [99].* E. purpurea* extract reduced the risk of respiratory complications by preventing virus-induced bacterial adhesion because it significantly reduced the expression of ICAM-1, fibronectin, and platelet activating factor receptor (PAFr) and consequently the adhesion of both bacterial strains [100].* E. purpurea* extract reduced the risk of respiratory complications through the inhibition of inflammation superstimulation (cytokine storms) by suppressing the expression of NFkB and possibly TLR-4 [100]. Moreover, in animal models,* Echinacea* restored serum cytokine levels, including interleukin-6 (IL-6), interleukin-10 (IL-10), and interleukin-17 (IL-17), as well as the mRNA expressions of these cytokines in the spleen [99]. This immunomodulatory effect of* Echinacea* has also been confirmed in a pilot study in healthy subjects considering the expression levels of the cytokines IL-2, IL-8, IL-6, and TNF- alfa in lymphomonocytes and in plasma samples measuring the mRNA and protein levels [101].

#### 3.4.3. Third Barrier: Adaptive Immunity

*Echinacea* treatment significantly increased the percentages of CD^4+^ and CD^8+^ T lymphocytes in the blood of rats [99]. Moreover, water-soluble extract from* Echinacea purpurea (L.) Moench* has dose-related adjuvant effects on human T cell cytokine responses characterized by enhancing and suppressive effects that are regulated by T cell density [102]. The mechanism of action involved modulatory effects indicating a possible role for water-soluble extract from* Echinacea purpurea (L.) Moench* (EchNWA) in enhanced Ca2+ mobilization and T cell activation. The authors underline that only the polysaccharide fraction was responsible for the immune modulatory effects described.

#### 3.4.4. *Echinacea* and Common Colds

A double-blind, randomized, placebo-controlled trial demonstrated that the combination of* Echinacea purpurea*, zinc, selenium, and vitamin C may alleviate exacerbation symptoms in 108 chronic-obstructive pulmonary disease (COPD) patients with acute upper respiratory tract infections (URTI) [45].* Echinacea* also seems to have the same synergistic effect in combination with* Justicia adhatoda* and* Eleutherococcus senticosus,* as demonstrated in a parallel-group, randomized, double-blinded, placebo-controlled trial, in which this combination of extracts exerted significant antitussive effects in acute upper respiratory tract infections (URI) [103].

Regarding RCT with* Echinacea* supplementation alone, various studies demonstrated that use of this plant may be a complementary treatment of respiratory tract infections. In a randomized, double-blind, placebo-controlled trial,* Echinacea* reduced the total number of cold episodes, cumulated episode days within the group, and pain-killer medicated episodes; inhibited virally confirmed colds; and especially prevented enveloped virus infections. It showed maximal effects on recurrent infections, and preventive effects increased with therapy compliance and adherence to the protocol [104]. The authors suggest a prophylactic intake of* E. purpurea* over 4 months to provide a positive risk to benefit ratio [104]. In 2012, a study demonstrated that a highly standardized extract from roots of* Echinacea angustifolia* with a specific phytochemical profile (presence of the complex polysaccharide IDN5405, the phenylethanoid echinacoside, and substantial lack of alkamides) could enhance the immune response subsequent to the influenza vaccination [105]. Another randomized, double-blind, double-dummy, multicenter, controlled clinical trial compared a new* Echinacea* formulation with a neuraminidase inhibitor, the gold standard treatment for influenza, and demonstrated the same effect [106]. The same authors explain other possible benefits of this natural treatment, such as lack of induction of drug resistance and complications [106]. A recent meta-analysis of randomized controlled trials indicated that* Echinacea* lowers the risk of recurrences and development of complications of respiratory tract infection, through antiviral and anti-inflammatory effects and the modulation of immune system [107]. However, in 2014 a Cochrane review on* Echinacea* for preventing and treating the common cold was published, demonstrating that results of individual prophylaxis trials consistently show positive (if nonsignificant) trends [33].

In conclusion, the use of this plant represents a complementary treatment of respiratory tract infections. Prophylactic treatment with* Echinacea* extracts (2400 mg/day for prevention and 4000 mg/day during acute stages of colds) over 4 months appeared to be beneficial for preventing/treating CC.

## 4. Conclusion

In European populations, adults have between 2 and 5 infections annually and children typically present 6 to 12 “colds” per year, and rates of symptomatic infections increase in the elderly [2]. Maintaining the immune defense system within a normal healthy state lowers the incidence of infection and/or lessens the severity of symptoms and/or shortens the duration of common colds. The immune system is an intricate network of specialized tissues, organs, cells, and chemicals protecting the host from infectious agents and other noxious insults. Although these defense mechanisms against invaders are very complex, they can be described as being organized in three main interactive clusters: physical barriers, and innate and adaptive immunity [9, 10]. The intakes of some nutrients and botanicals can significantly influence several components of immunity [26]. There are three nutrients that have specific EFSA scientific opinion on the substantiation of health claims related to vitamin D [27], vitamin C [28], zinc [29], and normal function of the immune system. Moreover, there is EFSA scientific opinion on the substantiation of health claims related to zinc [30] and to vitamin C [31] and maintenance of normal physical barriers, that is, the first immune system barriers. Finally, for vitamin C [32] and* Echinacea* [33], there are Cochrane reviews regarding the use of these two nutrients for preventing and treating the common cold.

Therefore, vitamin D, vitamin C, zinc, and* Echinacea* have pivotal roles of three main immunoreactive clusters (physical barriers, innate and adaptive immunity) in terms of prevention and treatment (shortening the duration and/or lessening the severity of symptoms) of common colds. The present narrative review demonstrated that current evidence of efficacy for zinc, vitamins D and C, and* Echinacea* is quite strong that CC patients may be encouraged to try them for preventing/treating their colds.

Regarding vitamin C, regular supplementation (1 to 2 g/day) has shown that vitamin C may reduce the duration (in adults by 8%, in children by 14%) and the severity of CC.

Considering zinc, supplementation may shorten the duration of colds by approximately 33%. CC patients may be instructed to try zinc within 24 hours of onset of symptoms.

Regarding vitamin D, the supplementation protected against CC overall. Baseline levels and age need to be considered. Patients who were deficient in vitamin D and those not receiving bolus doses experienced the most benefit.

As for* Echinacea*, the use of this plant represents a complementary treatment of respiratory tract infections. Prophylactic treatment with* Echinacea* extracts (2400 mg/day for prevention and 4000 mg/day during acute stages of colds) over 4 months appeared to be beneficial for preventing/treating CC.

## Figures and Tables

**Table 1:** Studies on zinc, immunity, and common cold presented in the narrative review.

Authors	Type of study	Subjects	Dosage	Results	Type of immunity Involved	Mechanism of action
Turner; 2001	Clinical study, randomized	Ninety-one volunteers	41 treated with active medication (consisted of 33 mM zinc gluconate) and 50 treated with placebo for 3 days were inoculated with rhinovirus and then were treated with study medication for an additional 6 days, single nasal spray of 120 mL per nostril, at ∼4-h intervals, 5 times each day	Zinc treatment had no effect on total symptom score, rhinorrhea, nasal obstruction, or the proportion of infected volunteers who developed clinical colds		

Mahalanabis et al.; 2004	Randomized, double-blind, placebo-controlled clinical trial	153 children aged 2–24	Four treatment: (1) zinc acetate (10 mg elemental Zn twice daily for 5 d) plus a placebo for vitamin A, (2) vitamin A as retinyl palmitate [10,000 *μ*g retinol equivalents (RE) twice daily for 4 d] plus a placebo for zinc, (3) zinc plus vitamin A according to the above schedule, or (4) placebo for zinc and for vitamin A	Zinc treatment significantly reduces duration of fever and very ill status in boys, but not in girls, with severe acute lower respiratory infection (ALRI)		Zinc as a micronutrient plays a key role at the catalytic sites of a wide range of enzymes and is critical to human growth, metabolism, and immune function. The diets of children in many developing countries are often deficient in zinc and a high phytate : zinc ratio in their diet reduces zinc bioavailability

Barnett et al.; 2016	A randomized double-blind, placebo-controlled trial	53 nursing home elderly (aged ≥65 y)	Supplementation with 30 mg Zn/d for 3 mo	The increase in serum zinc concentration is associated with the enhancement of T cell function	Adaptive immunity	Mainly because of an increase in the number of T cells and Zn improves T cell-mediated function by increasing the number of functional T cells in the periphery

Prasad et al.; 2007	Randomized, double-blind, placebo-controlled trial	Fifty healthy subjects of both sexes aged 55–87 y	Each day for 12 mo, subjects in the zinc-supplemented group received 1 capsule of zinc gluconate (15 mg elemental zinc) orally	The mean incidence of infections per subject in 12 mo was significantly lower in the zinc-supplemented group than in the placebo group. A significantly lower incidence of fever and a nonsignificant trend toward a lower incidence of the common cold were observed in the zinc-supplemented group than in the placebo group	Innate immunity	Zinc supplementation decreased not only the production of inflammatory cytokines but also that of oxidative stress marker: the increase of IL-2 production in zinc-deficient elderly subjects by increasing the gene expression of IL-2 and by a decrease in IL-10 production

Pinna et al.; 2002	Clinical study	8 healthy men	The subjects were given a controlled 3-d rotating diet that contained 4.6 mg Zn/d. Zn intakes were adjusted by giving Zn gluconate supplements. During baseline periods and repletion periods, subjects' Zn intakes totaled 13.7 mg/d. During restriction period, the subjects consumed 4.6 mg of highly available Zn	Changes in lymphocyte proliferation and IL-2R expression may be early markers of mild zinc deficiency	Innate immunity	Zinc restriction reduced peripheral blood mononuclear cells (PBMNC) proliferation at all mitogen concentrations tested except 10 mg/L. Dietary zinc restriction reduced IL-2R secretion by PBMNC stimulated at a suboptimal PHA concentration. The secretion of IFN-*γ* and TNF-*α* was unchanged throughout the study

Maggini et al.; 2012	Data from two preliminary, double-blind, randomized, placebo-controlled trials, parallel-group	Study 1 included 30 patients: 13 males and 17 females Study 2 enrolled 64 patients: 25 males and 39 females	1000 mg vitamin C plus 10 mg zinc	Supplementation with vitamin C and zinc may represent an efficacious measure		

Prasad et al.; 2008	Randomized, double-blind, placebo-controlled trial	Fifty ambulatory volunteers were recruited within 24 h of developing symptoms of the common cold	1 lozenge containing 13.3 mg of zinc (as zinc acetate) or placebo every 2-3 h while being awake	Mean durations of cold symptoms, cough, nasal discharge, and muscle ache were significantly decreased in the zinc group compared with the placebo group thanks to its anti-inflammatory and antioxidant properties	Innate immunity	The decrease in sIL-1ra and sTNF-R1 levels in the zinc group only suggests that zinc decreased the oxidative stress, resulting in decreased activation of monocytes and macrophages Zinc decreases ICAM-1 levels and induces the zinc-dependent transcription factor A20 in monocytes and macrophages, which inhibits NF-kB activation via the TNF-R-associated factor pathway Zinc is an inhibitor of NADPH oxidase, an enzyme that initiates the generation of free radicals; it is essential for superoxide dismutase and for generation of metallothionein, reduced the concentration of the oxidative stress markers, and inhibited the ex vivo induction of TNF-*α* and IL-1*β* mRNA in mononuclear cells; and it provided protection against TNF-*α*-induced NF-kB activation in isolated peripheral blood mononuclear cells

Bhandari et al.; 2002	Double masked, randomized placebo-controlled trial	2482 children aged 6 to 30 months	Daily elemental zinc, 10 mg to infants and 20 mg to older children or placebo for four months. Zinc gluconate	Zinc supplementation substantially reduced the incidence of pneumonia in children who had received vitamin A	Skin Innate immunity and adaptive immunity	Supplementation improves immune functions, including delayed cutaneous hypersensitivity, and increases the number of CD4 (helper) lymphocytes In experimental models zinc deficiency has been shown to impair cellular and humoral immune function

Bogden et al.; 1990	Double-blind partial crossover design	63 subjects (age 60–89)	3–6 month supply of placebo, 15 mg of Zn, or 100 mg Zn-capsules	Zinc had positive effects on one measure of cellular immune function but at the same time had an adverse effect on another measure of cellular immunity	Innate immunity	NK cell activity was enhanced, but, except for this cellular immune functions, were not significantly improved Delayed dermal hypersensitivity (DDH) increased in the study in placebo group and the suppression of the increase in DDH by zinc persisted

Mc Donald et al.; 2015	A randomized, double-blind, placebo-controlled clinical trial	2400 infants who were 6 wk of age and born to HIV-negative mothers in a low-malaria setting, Tanzania	Oral supplementation of MVs (vitamin B complex and vitamins C and E), zinc, zinc + MVs, or placebo for 18 mo. From the time of randomization to 6 mo of age, infants received 1 capsule/d, and from 7 mo of age to the end of follow-up, 2 capsules were provided daily The capsule contained 5 mg of zinc	Acute upper respiratory infections were significantly lower for infants supplemented with zinc than for those who did not receive zinc	Adaptive immunity	Mean CD4 T cell percentage was slightly but significantly higher among children who received zinc and MVs in comparison with placebo

Isbaniah et al.; 2010	Double-blind, randomized, placebo-controlled trial	Chronic obstructive pulmonary disease (COPD) patients with acute upper respiratory tract infection (URTI)—108 mostly male patients (age 40–81)	Treatment: EP (*Echinacea purpurea*) or EP + zinc, selenium, and ascorbic acid or placebo zinc = 10 mg	The combination of EP—zinc, selenium, and ascorbic acid—alleviates exacerbation symptoms caused by URTI in COPD	Innate and adaptive response	It seems that both selenium and zinc act on T lymphocytes, through modulating IL-2 secretion, its receptor expression, and sensitivity, as well as the thymulin activity which is required for the differentiation of CD4+ T cells. Zinc is included in the biosynthesis of leukotriene B4. Finally, the transcriptional repressor Gfi1, a zinger finger protein, was identified as a regulator of the innate immune response and to be essential for the development of macrophages-dependent cytokine production and granulocytes

Field et al.; 1987	Clinical study	15 female patients	Zinc supplements (50, 100, 150 mg)	Evidence in plasma and leucocyte zinc concentrations in an elderly population	Innate immunity	Decrease in granulocyte with high zinc doses probably thanks to the diminished phagocytic and chemotactic activity of polymorphonuclear cells

Wintergerst et al.; 2005	Review: a large number of randomized controlled intervention trials	Human	Daily intakes of 10–30 mg of zinc for children with infectious diseases	The authors explain that zinc and vitamin C play an important role in immune system: adequate intakes ameliorate symptoms and shorten the duration of respiratory tract infection, including the common cold, but there are discrepancies in trials that have been considered	Innate and adaptive immunity	Zinc is essential for the intracellular binding of tyrosine kinase to T cell receptors, which are required for T lymphocyte development and activation—lowered zinc status impairs cellular mediators of innate immunity such as phagocytosis by macrophages and neutrophils, natural killer cell activity, generation of the oxidative burst, and complement activity

Sanchez et al.; 2014	Randomized triple-blind community trial	301 children, 2–5 years of age	Children were distributed in three groups receiving zinc amino acid chelate, zinc sulfate, and placebo five days a week for 16 weeks 7 mg of zinc for children with 2-3 years and 9,45 mg of zinc for children with 4-5 years	Zinc amino acid chelate had a better effect on reducing the incidence of acute respiratory infection in children	Innate immunity	Probably thanks to the production of interferon and the modulation of inflammatory cytokines

Martinez- Estevez et al.; 2016	12-month randomized controlled trial, triple-blind	355 children underwent randomization, with 174 assigned to the zinc supplementation group and 181 to the control group	Children in the active supplementation group received 5 mg of zinc oxide plus 525 mg of calcium carbonate plus 70 UI of vitamin D3 (Kid Cal), and children in the nonsupplemented control group received 525 mg of calcium carbonate plus 70 UI of vitamin D3	Decreased the incidence of URTI	Innate immunity	Among the various mechanisms involved in the antiviral effects of zinc, the ICAM-1 receptor blocking has been considered as one of the most important actions

Brooks et al.; 2004	Double-blind placebo-controlled trial	270 children (age 2–23 months)	Elemental zinc, 20 mg per day, or placebo	Zinc accelerates recovery from severe pneumonia in children and could help reduce antimicrobial resistance by decreasing multiple antibiotic exposures and lessen complications and deaths where second line drugs are unavailable	Innate immunity	Zinc has role in the acute phase response mediated by cytokines during acute infection, helping to boost the body's immune response through a defense cascade, beginning with mobilization and sequestration of zinc to metallothionein-rich tissue, rapid upregulation of immune defense specific protein synthesis, activation of immune defense activity such as macrophages, lymphocytes, and NK cells and antibody-dependent cytotoxicity

Sazawal et al.; 1997	A double-blind, randomized controlled trial	Children (zinc 38, control 48)	Zinc gluconate to provide elemental zinc 10 mg daily and 20 mg during diarrhea	Zinc supplementation improves cellular immune status: regarding cell-mediated immunity (CMI), the percentage of anergic or hypoergic children (using induration score) decreased from 67% to 47% in the zinc group. The zinc group had a significantly higher rise in the geometric means of CD3, CD4, and CD4/CD8 ratio with no difference in CD8 and CD20. The rise in CD4 was significantly higher in the zinc as compared to the control group	Adaptive immunity	The observation of an increase in the number of circulating T lymphocytes, especially CD4 cells, after zinc supplementation may be explained by direct effect of zinc ion on the lymphocyte membrane affecting maturation and differentiation of T lymphocytes or by a stimulation of thymus endocrine function. Once T lymphocytes leave the thymus their differentiation and maturation are thought to be regulated by zinc-thymulin and deficiency of zinc-thymulin has been associated with secondary cellular immune deficiency and with immune senescence. Thus terminal deoxynucleotidyl transferase or zinc-thymulin has been suggested as possible mechanism by which zinc may be affecting T cell development and function

Sempertegui et al.; 1996	A randomized double-blind placebo-controlled trial	50 children (age 12–59 months old)	10 mg of zinc sulfate or placebo	After treatment (on day 60), the cutaneous delayed-type hypersensitivity (DTH) was higher in treated group; the incidence of fever, cough, and upper respiratory tracts secretions was lower in S group, but after 120 days the incidence of fever and upper respiratory tracts secretions was the same in both groups, but the incidence of cough was higher in S group	Innate immunity	The mechanism is not clear, probably improving cellular immunity

Bogden et al.; 1988	Clinical study	103 elderly subjects (age 60–89 years)	Three treatments: placebo, or 15 mg zinc/day or 100 mg/day for 3 months	In a subgroup (34,3%), Zn administration enhanced delayed dermal hypersensitivity (DDH)	Skin, innate immunity	cellular immunity will not be enhanced by Zn supplementation, but they argued that cellular immunity in subgroups of elderly people will be improved by Zn supplementation

Fortes et al.; 1998	A double-blind, randomized, controlled trial	178 elderly patients	4 treatments: (1) vit. A (800 *μ*g retinol palmitate), (2) zinc (25 mg as zinc sulfate), (3) zinc + vit. A (800 *μ*g retinol palmitate and 25 mg of zinc sulfate), (4) placebo	Zinc supplementation improves cell-mediated immune response, because it increases the number of CD4+DR+T cells and cytotoxic T lymphocytes	Immune and adaptive response	Effect on thymulin levels that promotes T cells functions, including suppressor function and interleukin-2 production. Zn prevents the negativity effects of vit. A on immunity

Turk et al.; 1998	Clinical study	26 patients in hemodialysis and 11 healthy patients (HP) were vaccinated with multivalent influenza vaccine (MIV)	Supplementation with 120 mg of ZnSO4	Zn supplementation could not restore the immune parameters and enhance antibody response to MIV in HP		

Wieringa et al.; 2010	Randomized, double-blind, controlled trial	229 pregnant women with a gestational age <20 weeks, and infants and women were followed up monthly until the infants were 6 months old	In addition to iron (30 mg as ferrous fumarate) and folic acid (0,4 mg), one group of women received *β*- carotene (4,5 mg), one group zinc (30 mg as zinc sulfate), and one group *β*- carotene plus zinc (4,5 and 30 mg, resp.)	Maternal supplementation with zinc and *β*-carotene affected the newborn's immune development, but only zinc supplementation affects morbidity in the infants	Innate immunity	Zinc gives higher interleukin-6 production

Erickson et al.; 2000	Review	Humans	Supplementation such as zinc, selenium, iron, copper, b-carotene, vitamins A, C, and E, and folic acid	Micronutrients have an important role in immunity	Innate immunity	Zinc enhances natural killer cell functions; zinc supplementation directly induced cytokine production, predominantly IL-1, IL-6, and TNF-a, by mononuclear cells in vitro

Maggini et al.; 2007	Review	Humans		Inadequate intake and status of vitamins and trace elements may lead to suppressed immunity, which predisposes to infections and aggravates undernutrition. Therefore, supplementation with these selected micronutrients (including zinc) can support the body's natural defense system by enhancing all three levels of immunity	Skin innate and adaptive immunity	It is involved in the cytosolic defense against oxidative stress (superoxide dismutase activity) and is an essential cofactor for thymulin which modulates cytokine release and induces proliferation. It helps to maintain skin and mucosal membrane integrity and increases cellular components of innate immunity (e.g., phagocytosis by macrophages and neutrophils, NK cell activity, generation of oxidative burst, DTH activity), antibody responses, and the numbers of cytotoxic CD8þT cells (Th1 response)

Calder and Kew; 2002	Review			Increasing intakes of some nutrients above habitual and recommended levels can enhance some aspects of immune function; low plasma Zn levels predicted the subsequent development of lower respiratory tract infections	Skin innate and adaptive immunity	In patients with Zn deficiency related to sickle cell disease, natural killer cell activity is decreased. Zinc supplementation increases thymus size, and topical application of Zn improves the DTH response in the area of skin on which the application was made Zn administration to preterm-low-birth-weight infants increases the number of circulating T lymphocytes and their proliferation and a 5 mg Zn/d increases measures of cell-mediated immune function

Singh and Das; 2013	Review	Humans	There is a significant reduction in the duration of cold at a dose of ≥75 mg/day	Zinc administered within 24 hours of onset of symptoms reduces the duration of common cold symptoms in healthy people	Enhancement of innate as well as acquired immunity	Not clear

Hojyo and Fukada; 2016	Review			Zn has role in dendritic cells (that are important to present the peptide-MHC-II complex on their cell surface to antigen-specific CD4+ helper T (Th) cells to initiate immune responses) because a reduction in Zn is required for proper antigen presentation via MHC-II to elicit adaptive immune responses Zn deficiency is characterized by immunodeficiency with thymic atrophy and lymphopenia Zn controls antibody-mediated humoral immune responses and ZIP10-Zn has a role in early B-cell development and the maintenance of mature B cells ZIP10-Zn signaling may control fate decisions in lymphocyte progenitors under physiological conditions and exacerbate malignancy under pathological conditions, according to the highly regulated pattern of ZIP10 expression	Innate and adaptive immunity	Zn facilitates the endocytosis of MHC-II but inhibits the trafficking of MHC-II from the lysosome/endosome compartments to the plasma membrane the ZnD-induced thymic atrophy could result from the combination of increased glucocorticoid levels, an impairment of thymulin activity, and impaired cell-intrinsic survival function ZIP10-Zn signaling regulates the expression of CD45R while simultaneously (and indirectly) enhancing the CD45R PTPase activity through a Zn-dependent process rather than by a direct effect on PTPase activity The effect on development could be explained by glucocorticoids and ZIP10 maintains mature B cells through a LYN-independent mechanism ZIP10-Zn signaling inhibits the apoptosis induced by activated caspases and promotes pro-B-cell survival in a cell-autonomous manner Cytokine stimulation (the first signal) activates the JAK-STAT pathway (the second signal), which further induces ZIP10 expression and eventually generates ZIP10-Zn signals (the third signal)

**Table 2:** Studies on vitamin D, immunity, and common cold presented in the narrative review.

Authors	Type of study	Subjects	Dosage	Results	Type of immunity involved	Mechanism of action
Bergman et al.; 2015	A randomized, placebo-controlled, and double-blinded study	The per protocol population (*n* = 124 patients aged 18 to 75 years), who completed the study, consisted of *n* = 62 vitamin D treated and *n* = 62 placebo treated patients.	*n* = 62 oral vitamin D3 (4000 IU/day for 1 year) treated and *n* = 62 oil (Miglyol) placebo treated patients. Follow-up: 12 months.	Vitamin D supplementation increased the probability of staying free of respiratory tract infections (RTI) during the study year (RR 0.64, 95% CI 0.43–0.94). Further, the total number of RTIs was also reduced in the vitamin D group (86 RTIs) versus placebo (120 RTIs; *p* = 0.05). Finally, the time to the first RTI was significantly extended in the vitamin D group (HR 1.68, 95% CI 1.03–2.68, *p* = 0.0376).		The role of vitamin D in respiratory tract infections is still not clear, despite several large RCTs in the area. This can probably be explained by the large heterogeneity in these randomized controlled trials (RCTs).

Bock et al.; 2011	A double-blind, placebo-controlled trial	59 healthy adult subjects (49% females).	Subjects received oral vitD3 (140,000 IU oleovitD3, monthly) or placebo (almond oil) for a period of 3 months.	% regulatory T cells (Tregs) increased significantly only in the vit D group. A short time high-dose vitD3 supplementation significantly increased the frequency of Tregs, but did not further improve *β*-cell function in apparently healthy subjects.	Adaptive immunity: T cells and *β* cell function	The immunomodulatory potential of vit D might be an important mechanistic link for the association of vit D and T1D.

Goodall et al.; 2014	Double-blind clinical trial	600 participants (≥17 years), 471 (78.5%) completed all surveys while 43 (7.2%) completed none.	Participants were randomized to receive a container with eight capsules of either 10,000 IU of active vitamin D3 or identical placebo. Students are randomized into 4 treatment arms: (1) vitamin D3 and gargling, (2) placebo and gargling, (3) vitamin D3 and no gargling, and (4) placebo and no gargling.	Of 600 participants, 471 (78.5%) completed all surveys while 43 (7.2%) completed none; 150 (25.0%) reported clinical URTI. Seventy participants (23.3%) randomized to vitamin D3 reported clinical URTI compared to 80 (26.7%) randomized to placebo (RR: 0.79, CI95: 0.61–1.03, *p* = 0.09). Eighty-five participants (28.3%) randomized to gargling reported clinical upper respiratory tract infection (URTI) compared to 65 participants (21.7%) randomized to the no gargling arm (RR: 1.3, CI95: 0.92–1.57, *p* = 0.19). Laboratory testing identified 70 infections (46.7 per 100 URTIs). Vitamin D3 treatment was associated with a significantly lower risk for laboratory confirmed URTI (RR: 0.54, CI95: 0.34–0.84, *p* = 0.007) and with a significantly lower mean viral load measured as log10 viral copies/mL (mean difference: −0.89, CI95: −1.7, −0.06, *p* = 0.04). Fewer students assigned to gargling experienced laboratory confirmed URTI; however this was not statistically significant (RR: 0.82, CI95: 0.53–1.26, *p* = 0.36).		Vitamin D3 is a promising intervention for the prevention of URTI. Vitamin D3 significantly reduced the risk of laboratory confirmed URTI and may reduce the risk of clinical infections.

Camargo et al.; 2012	Double-blind clinical trial	744 Mongolian children from 21 third- and fourth-grade classrooms (9-10 years old), but this analysis focused on a subset of 247 children who were assigned to daily ingestion of unfortified regular milk or milk fortified with 300 IU of vitamin D3. 50% of children, who drank milk, were male. 54% of children, who drank milk vitamin D fortified were male.	The Blue Sky Study examined 5 approaches to improve the vitamin D status of Mongolian schoolchildren: (1) 300 IU of vitamin D3 daily in Mongolian milk (*n* = 143); (2) 300 IU of vitamin D3 daily in US milk (*n* = 143); (3) 300 IU of vitamin D3 daily in a milk substitute (*n* = 147); (4) 300 IU of vitamin D3 in a daily pill (*n* = 112); and (5) a total of 13,700 IU of vitamin D3 in pills given over the first 7 days of study (*n* = 95). Follow-up: winter (January–March).	At baseline, the median serum 25(OH)D level was 7 ng/mL (interquartile range: 5–10 ng/mL). At the end of the trial, follow-up was 99% (*n* = 244), and the median 25(OH)D levels of children in the control versus vitamin D groups were significantly different (7 versus 19 ng/mL; *p*= 0.001). Compared with controls, children receiving vitamin D reported significantly fewer ARIs during the study period (mean: 0.80 versus 0.45; *p* = 0.047), with a rate ratio of 0.52 (95% confidence interval: 0.31–0.89). Adjusting for age, gender, and history of wheezing, vitamin D continued to halve the risk of ARI (rate ratio: 0.50 [95% confidence interval: 0.28–0.88]). Similar results were found among children either below or above the median 25(OH)D level at baseline (rate ratio: 0.41 versus 0.57; *p* for interaction = .27).		Vitamin D supplementation significantly reduced the risk of ARIs in winter among Mongolian children with vitamin D deficiency.

Urashima et al.; 2010	A multicenter, randomized, double-blind, placebo-controlled, parallel-group trial	430 schoolchildren (56% were male) aged 6–15 y, with or without underlying diseases, were eligible and asked to participate in the study by the pediatricians in charge of the outpatient clinics.	The participants were asked to take 3 tablets twice daily, total: 1200 IU vitamin D3 (217 children) or placebo (213 children). The accrual period was from December 1, 2008, to March 31, 2009.	Influenza A occurred in 18 of 167 (10.8%) children in the vitamin D3 group compared with 31 of 167 (18.6%) children in the placebo group [relative risk (RR), 0.58; 95% CI: 0.34, 0.99; *p* = 0.04]. The reduction in influenza A was more prominent in children who had not been taking other vitamin D supplements (RR: 0.36; 95% CI: 0.17, 0.79; *p* = 0.006) and who started nursery school after age 3 y (RR: 0.36; 95% CI: 0.17, 0.78; *p* = 0.005). In children with a previous diagnosis of asthma, asthma attacks as a secondary outcome occurred in 2 children receiving vitamin D3 compared with 12 children receiving placebo (RR: 0.17; 95% CI: 0.04, 0.73; *p* = 0.006).		Vitamin D3 supplementation during the winter may reduce the incidence of influenza A, especially in specific subgroups of schoolchildren.

Şişmanlar et al.; 2016	Clinical trial	Sixty-three children aged between six months and five years with lower respiratory infections and 59 age-matched children who had no history of respiratory symptoms in the last month and no accompanying chronic disease.	Follow-up: December 2010 and February 2011. 25 (OH) vitamin D level of <20 ng/mL was considered vitamin D deficiency, a level of 20–30 ng/mL was considered vitamin D insufficiency, a level of >30 ng/mL was considered normal, and a level of >150–200 ng/mL was considered intoxication.	No significant correlation was found between vitamin D levels and lower respiratory tract infection in terms of disease and its severity. However, it was found that vitamin D deficiency/insufficiency was observed with a high rate in all children included in the study.		Although no correlation was found between vitamin D level and lower respiratory tract infection, it is recommended that vitamin D level should be measured in children with lower respiratory tract infection and vitamin D supplementation should be given to all children especially in winter months based on the fact that the level of vitamin D was lower than normal in approximately half of the children included in the study and considering the effects of vitamin D on infections, pulmonary functions, and immunity.

Li-ng et al.; 2009	A 3-month prospective, randomized, double-blind, placebo-controlled trial of vitamin D3 supplementation in ambulatory adults	162 adults (18–80 years old) were randomized. Male: 17 active, 13 placebo. Female: 61 active, 57 placebo.	84 patients received 50 *μ*g/d vitamin D3 and 78 patients received placebo. Follow-up: March–June 2007.	There were no significant differences between the active and placebo patients at baseline. The baseline 25-OHD levels ranged from 16 to 156 nmol/l with mean level of 63.7 ± 28.7 nmol/l in the study population. At baseline, 23% of the active patients exceeded 75 nmol/l.		

Bischoff-Ferrari et al.; 2012	Clinical trial	20 white postmenopausal women, 50 to 70 years of age, in general good health with an average 25(OH)D level of 13.2 ± 3.9 ng/mL (mean ± SD) and a mean age of 61.5 ± 7.2 years were randomized to either 20 mg of HyD or 20 mg (800 IU) of vitamin D3 per day in a double-blind manner.	Participants attended one screening visit and 14 clinical visits during a 4-month trial period. Women who passed eligibility criteria signed informed consent and were randomly assigned to one of four groups: 20 mg HyD daily, 20 mg (800 IU) vitamin D3 daily, 140 mg HyD weekly, or 140 mg (5600 IU) vitamin D3 weekly. All supplements were taken orally. We randomized five participants to each treatment group. The dose of vitamin D3 was chosen to compare to the current standard for vitamin D3 (20 mg(1/4)800 IU/d).	Mean 25(OH)D levels increased to 69.5 ng/mL in the HyD group. This rise was immediate and sustained. Mean 25(OH)D levels increased to 31.0 ng/mL with a slow increase in the vitamin D3 group. Women on HyD compared with vitamin D3 had 2.8-fold increased odds of maintained or improved lower extremity function (odds ratio [OR](1/4)2.79; 95% confidence interval [CI], 1.18–6.58) and a 5.7-mmHg decrease in systolic blood pressure (*p*(1/4)0.0002). Both types of vitamin D contributed to a decrease in five out of seven markers of innate immunity, significantly more pronounced with HyD for eotaxin, IL-12, MCP-1, and MIP-1 b. There were no cases of hypercalcemia at any time point. Twenty micrograms (20 mg) of HyD per day resulted in a safe, immediate, and sustained increase in 25(OH)D serum levels in all participants, which may explain its significant benefit on lower extremity function, systolic blood pressure, and innate immune response compared with vitamin D3.	innate immune response	The study shows that 20 mg HyD is significantly more efficient and more rapid in shifting healthy postmenopausal women into a desirable 25(OH)D serum level of at least 30 ng/mL compared to 800 IU (20 mg) vitamin D3.

Charan et al.; 2012	A systematic review and meta-analysis	Humans Adults (18–80 year, 18–28 men, and postmenopausal women) and children (1–3 year, 6–15 year). The population number is not reported.	Dose of vitamin D used in these clinical trials ranged from 400 IU/day to 2000 IU/day. In one clinical trial single parenteral dose of vitamin D was given (100000 IU).	Events of respiratory tract infections were significantly lower in vitamin D group as compared to control group [odds ratio = 0.582 (0.417–0.812) *p* = 0.001] according to random model. On separate analysis of clinical trials dealing with groups of children and adults, beneficial effect of vitamin D was observed in both, according to fixed model [odds ratio = 0.579 (0.416–0.805), *p* = 0.001 and odd ratio = 0.653 (0.472–0.9040), *p* = 0.010 resp.].	Innate and adaptive immunity	It is believed that vitamin D increases the production of natural antibodies. Vitamin D is also known to strengthen the immunity by inducing monocyte differentiation and inhibiting lymphocyte proliferation. It is also postulated that vitamin D enhances the phagocytic activity of macrophages.

Chen et al.; 2016	Clinical trial	99 women with a history of two or more successive miscarriages. A total of 35 patients constituted the vitamin D normal group (VDN) (age 33.6 y ± 3.9), and 51 patients were included in the vitamin D insufficiency group (VDI) (age 32.4 y ± 3.9), and 13 vitamin D deficiency patients in VDD (age 33.0 y ± 4.4).	Patients with recurrent miscarriage (RM) were supplemented with 0.5 *μ*g/day of 1,25(OH)2D for 2 months and then peripheral blood cells.	The percentage of CD19+ B cells and NK cytotoxicity at an effector-to-target cell (E : T) ratio of 50 : 1, 25 : 1, and 12.5 : 1 were significantly higher in the vitamin D insufficiency group (VDI) than in the vitamin D normal group (VDN) (*p* < .05 each). The proportion of TNF-*α*-expressing Th cells was significantly higher in the vitamin D deficiency group (VDD) than in VDN (*p* < .05). However, there were no significant differences between VDI and VDD. This dysregulation was significantly reduced with 1,25(OH)2D supplementation.	Innate and adaptive immunity: cell-mediated immunity	It was found that the percentage of peripheral blood CD19+ B cells, the percentage of TNF-*α*- producing Th cells, and NK cytotoxicity at all E : T ratios were dramatically reduced under 1,25(OH)2D supplementation. it has been shown that 1,25(OH)2D could downregulate several genes associated with TNF-*α*, including proteins involved in the transcription of TNF-*α*, one of its primary receptors, and TNF-*α* itself. Vitamin D was also able to depolarize perforin expression in the cytoplasm, which reduced the NK cytotoxicity in RM patients.

Dankers et al.; 2017	Review			The review discussed the effect of vitamin D which is the modulation of the immune system. The review discusses the current knowledge about the molecular mechanisms underlying the immunomodulatory effects of vitamin D and how these advances can be used in the treatment of autoimmune diseases.	Autoimmunity, innate and adaptive immunity	

De Gruijl and Pavel; 2012	Randomized clinical study	105 student volunteers (18–30 years of age) divide in 3 groups.	The participants were randomized to 3 groups: (A) subjected to 3 times a week sub-sunburn sunbed exposure (*n* = 35), (B) daily vitamin D supplementation, 1000 IU (*n* = 37), and (C) a control group without any intervention (*n* = 33). The mean serum level of 25-hydroxyvitamin D (25(OH)D) dropped from 62 to 55 nmol l^−1^ in group C, while these levels rose from 62 to 109 and from 58 to 93 nmol l^−1^ in groups A and B.	Although fewer colds occurred in the groups (A) and (B) compared with control group (C), the difference was not significant. The initial 25(OH)D levels in the volunteers that caught a cold (*n* = 61) were not significantly different from those who did not (*n* = 44): mean of 61 (SD 21) versus 61 (SD 20) nmol l^−1^ (nor were the final levels different: mean of 86 (SD 31) versus 86 (SD 32) nmol l^−1^).	Innate immunity and skin barrier	The study shows sub-sunburn sunbed treatment to be effective in tanning and in increasing the 25(OH)D serum level, more so than oral vitamin D supplementation by 1000 IU per day. Despite earlier results suggesting a possible beneficial effect on colds, this 8-week mid-winter course of sunbed exposures has, however, no appreciable effect on colds.

de Sá Del Fiol et al.; 2015	Review	Humans.		This study aimed to review recent clinical and epidemiological studies conducted in adults and children and to evaluate the functional role of vitamin D in respiratory infections. The evaluated studies show an important immunomodulatory role of vitamin D, which reduces the incidence and risk of URTIs (upper respiratory tract infections), both in children and in adults. Combating URTIs can be done prophylactically, associating the use of vaccines against *Streptococcus pneumoniae* with strengthening the immune system through supplementation with vitamin D.	Innate and adaptive immune system	Vitamin D appears to combat infection via multiple mechanisms. It has a direct influence on the production of cathelicidin, which may lead to increased susceptibility to viruses and bacteria, and it influences cytokine profiles during infection via the innate and adaptive immune system.

Denlinger et al.; 2016	A clinical trial: the AsthmaNet VIDA (Vitamin D Add-on Therapy Enhances Corticosteroid Responsiveness) trial	408 adult patients.	Patients are randomized to receive placebo or cholecalciferol (100,000 IU load plus 4,000 IU/d) for 28 weeks as add-on therapy.	A total of 203 participants experienced at least one cold. Despite achieving 25-hydroxyvitamin D levels of 41.9 ng/ml (95% confidence interval [CI], 40.1–43.7 ng/ml) by 12 weeks, vitamin D supplementation had no effect on the primary outcome: the average peak WURSS-21 scores (62.0 [95% CI, 55.1–68.9; placebo] and 58.7 [95% CI, 52.4–65.0; vitamin D]; *p* = 0.39). The rate of colds did not differ between groups (rate ratio [RR], 1.2; 95% CI, 0.9–1.5); however, among African Americans, those receiving vitamin D versus placebo had an increased rate of colds (RR, 1.7; 95% CI, 1.1–2.7; *p* = 0.02). This was also observed in a responder analysis of all subjects achieving vitamin D sufficiency, regardless of treatment assignment (RR, 1.4; 95% CI, 1.1–1.7; *p* = 0.009).	Epithelium innate immunity	The authors hypothesized but did not observe that achieving vitamin D sufficiency would allow for enhanced responsiveness to ICS (the daily dose of inhaled corticosteroid) with respect to lung function owing to the ability of vitamin D to influence steroid metabolism. Conversely, it is also possible that the change in ICS doses during the protocol influenced vitamin D metabolism and/or the expression of the vitamin D receptor and binding protein, so it is possible that we did not give enough vitamin D and that 25(OH)D levels in the serum do not reflect the changes relevant to airway epithelium innate immunity.

Erickson et al.; 2000	Review			Micronutrients such as zinc, selenium, iron, copper, b-carotene, vitamins A, C, and E, and folic acid can influence several components of innate immunity. Selected micronutrients play an important role in alteration of oxidant-mediated tissue injury, and phagocytic cells produce reactive oxidants as part of the defense against infectious agents.	Innate immunity	Deficiencies in zinc and vitamins A and D may reduce natural killer cell function, whereas supplemental zinc or vitamin C may enhance their activity.

Hewison; 2012	Overview			It is now clear that cells from the immune system contain all the machinery needed to convert 25-hydroxyvitamin D to active 1,25- dihydroxyvitamin D, and for subsequent responses to 1,25-dihydroxyvitamin D. Such mechanisms are important for promoting antimicrobial responses to pathogens in macrophages and for regulating the maturation of antigen-presenting dendritic cells. The latter may be a key pathway by which vitamin D controls T lymphocyte (T cell) function. However, T cells also exhibit direct responses to 1,25-dihydroxyvitamin D, notably the development of suppressor regulatory T cells.	Innate and adaptive immunity	Vitamin D is a key factor linking innate and adaptive immunity, and both of these functions may be compromised under conditions of vitamin D insufficiency.

Gupta et al.; 2016	A randomized control trial	38 adults with vitamin D deficiency and untreated pre- or early stage I hypertension were included.	Participants are randomized to either low- (400 IU daily) or high- (4000 IU daily) dose oral vitamin D3 for 6 months.	Treatment with 4000 IU of vitamin D3 decreased intracellular CD4+ ATP release by 95.5 ng/ml (interquartile range, −219.5 to 105.8). In contrast, 400 IU of vitamin D3 decreased intracellular CD4+ ATP release by 0.5 ng/ml (interquartile range, −69.2 to 148.5). In a proportional odds model, high-dose vitamin D3 was more likely than low-dose vitamin D3 to decrease CD4+ ATP release (odds ratio, 3.43; 95% confidence interval, 1.06–1.11).	Adaptive immunity: cell-mediated immunity	Treatment with high-dose vitamin D3 reduces CD4 T cell activation, providing direct human data that vitamin D may influence cell-mediated immunity (CMI). These findings offer a mechanistic correlation for the potential influence of vitamin D on the course of immune-mediated disorders.

Laaksi et al.; 2010	A placebo-controlled double-blinded study	164 voluntary young Finnish men (18–28 years of age).	The subjects were randomly assigned to the intervention group, which received 400 IU (10 mg) vitamin D3 daily, or the control group, which received placebo for 6 months.	After daily supplementation, the mean serum 25(OH)D concentrations (±SD) were 71.6 ± 22.9 nmol/L (*n* = 58) in the intervention group and 51.3 ± 15.5 nmol/L (*n* = 50) in the placebo group (*p* < .001). The number of days of absence from duty due to respiratory tract infection did not differ between groups. Mean number of absence days (±SD) was 2.2 ± 3.2 days in the intervention group and 3.0 ± 4.0 days in the placebo group (*p* < 096). There was an effect during the first 6 weeks of the study, with a mean (±SD) of 0.7 ± 2.1 absence days in the intervention group and 1.4 ± 2.6 absence days in the placebo group (*p* < 060).	Innate immunity	

Maggini et al.; 2007	Review			The vitamins A, B6, B12, C, D, and E; folic acid; and the trace elements iron, zinc, copper, and selenium work in synergy to support the protective activities of the immune cells. Finally, all these micronutrients, with the exception of vitamin C and iron, are essential for antibody production. Overall, inadequate intake and status of these vitamins and trace elements may lead to suppressed immunity, which predisposes one to infections and aggravates malnutrition.	Innate, adaptive immunity and autoimmunity	Vitamin D and especially its biologically active metabolite 1,25-dihydroxycholecalciferol (1,25(OH)2D3) act as powerful immunoregulators. The discovery of significant quantities of vitamin D receptors in monocytes, macrophages, and thymus tissue suggests a specific role of vitamin D and its metabolites in the immune system. Most cells of the immune system except B cells express vitamin D receptors.

Martineau et al.; 2017	Systematic review and meta-analysis of individual participant data (IPD) from randomized controlled trials	Total 11321 participants, aged 0 to 95 years.	Benefit was greater in those receiving daily or weekly vitamin D without additional bolus doses (NNT = 20), and the protective effects against acute respiratory tract infection in this group were strongest in those with profound vitamin D deficiency at baseline (NNT = 4).	Vitamin D supplementation reduced the risk of acute respiratory tract infection among all participants (adjusted odds ratio 0.88, 95% confidence interval 0.81 to 0.96; *p* for heterogeneity < 0.001). In subgroup analysis, protective effects were seen in those receiving daily or weekly vitamin D without additional bolus doses (adjusted odds ratio 0.81, 0.72 to 0.91) but not in those receiving one or more bolus doses (adjusted odds ratio 0.97, 0.86 to 1.10; *p* for interaction = 0.05). Among those receiving daily or weekly vitamin D; protective effects were stronger in those with baseline 25-hydroxyvitamin D levels < 25 nmol/L (adjusted odds ratio 0.30, 0.17 to 0.53) than in those with baseline 25-hydroxyvitamin D levels ≥ 25 nmol/L (adjusted odds ratio 0.75, 0.60 to 0.95; *p* for interaction = 0.006).		

Mayan et al.; 2015	Clinical trial	82 adolescent swimmers for serum 25(OH)D and TREC (T cell receptor excision circles) concentrations; it was found that 55 had vitamin D insufficiency.	Fifty-five participants (67%) had vitamin D insufficiency and comprised an interventional study group, where subjects were randomized to receive supplementation of either vitamin D3 (2000 IU/day) in liquid drops or a placebo. Subjects who were vitamin D sufficient did not receive any supplementation or treatment. Randomized supplementation of either vitamin D3 or placebo was given for 12 winter weeks.	TREC concentrations decreased with the participants' age (*r* = −0.346, *p* = 0.003), with no significant between-gender difference. TREC concentrations did not materially differ among subjects with normal, insufficient, or deficient vitamin D status and were not affected by vitamin D supplementation. No significant correlations were found between TREC levels, or their changes during the study period, and mean upper respiratory infections (URI) severity or duration.	Adaptive immunity	The authors found no significant correlation between vitamin D and TREC levels, either before or after supplementation, suggesting that the immunomodulatory effects of vitamin D are not exerted directly on the thymus gland. Indeed, several other mechanisms by which vitamin D may influence T cell function have been proposed, including direct endocrine effects on T cells mediated via systemic calcitriol, direct intracrine conversion of 25(OH)D to calcitriol by T cells, direct paracrine effects of calcitriol on T cells following conversion of 25(OH)D to calcitriol by monocytes or dendritic cells, and indirect effects on antigen presentation to T cells mediated via localized antigen-presenting cells affected by calcitriol.

Schwalfenberg; 2011	Review			This review looks at the critical role of vitamin D in improving barrier function, production of antimicrobial peptides including cathelicidin and some defensins, and immune modulation. The function of vitamin D in the innate immune system and in the epithelial cells of the oral cavity, lung, gastrointestinal system, genitourinary system, skin, and surface of the eye is discussed.	Epithelial barrier and innate immunity	

Murdoch et al.; 2012	Double-blind, placebo-controlled trial	322 healthy adults.	Participants were randomly assigned to receive an initial dose of 200,000 IU oral vitamin D3, then 200,000 IU 1 month later, then 100,000 IU monthly (*n* = 161), or placebo administered in an identical dosing regimen (*n* = 161), for a total of 18 months.	The mean baseline 25-OHD level of participants was 29 (SD, 9) ng/mL. Vitamin D supplementation resulted in an increase in serum 25-OHD levels that was maintained at greater than 48 ng/mL throughout the study. There were 593 upper respiratory tract infections (URTI) episodes in the vitamin D group and 611 in the placebo group, with no statistically significant differences in the number of URTIs per participant (mean, 3.7 per person in the vitamin D group and 3.8 per person in the placebo group; risk ratio, 0.97; 95% CI, 0.85–1.11), number of days of missed work as a result of URTIs (mean, 0.76 days in each group; risk ratio, 1.03; 95% CI, 0.81–1.30), duration of symptoms per episode (mean, 12 days in each group; risk ratio, 0.96; 95% CI, 0.73–1.25), or severity of URTI episodes.		

Bartley; 2010	Review			Vitamin D is involved in the production of defensins and cathelicidin-antimicrobial peptides that provide a natural defense against potential microbiological pathogens. Vitamin D supplementation increases cathelicidin production. Low vitamin D levels are associated with an increased incidence of upper respiratory tract infections.	Innate immunity	

Jorde et al.; 2016	Randomized controlled trial	Five hundred and eleven subjects with prediabetes.	Participants are randomized to vitamin D3 (20,000 IU per week) versus placebo for five years. Two hundred and fifty-six subjects received vitamin D and 255 placebo. One hundred and sixteen subjects in the vitamin D and 111 in the placebo group completed the five-year study.	Mean baseline serum 25-hydroxyvitamin D (25(OH)D) level was 60 nmol/. Eighteen subjects in the vitamin D group and 34 subjects in the placebo group reported urinary tract infections (UTI) during the study (*p* < 0.02), whereas no significant differences were seen for respiratory tract infections (RTI). The effect on UTI was most pronounced in males. The effect of vitamin D on UTI was unrelated to baseline serum 25(OH)D level.		

Kamen and Tangpricha; 2010	Review			The importance of vitamin D in the regulation of cells of the immune system has gained increased appreciation over the past decade with the discovery of the vitamin D receptor (VDR) and key vitamin D metabolizing enzymes expressed by cells of the immune system.	Innate and adaptive immunity	The hormonal form of vitamin D upregulates antimicrobial peptides, namely, cathelicidin, to enhance clearance of bacteria at various barrier sites and in immune cells. Vitamin D modulates the adaptive immune system by direct effects on T cell activation and on the phenotype and function of antigen-presenting cells (APCs), particularly of DCs. The purpose of this manuscript is to review the molecular and clinical evidence for vitamin D as a modulator of the innate and adaptive immune system.

Bolland et al.; 2017	Editorial		Authors consider that current evidence does not support the use of vitamin D supplementation to prevent disease, except for those at high risk of osteomalacia, currently defined as 25-hydroxyvitamin D levels less than 25 nmol/L.	In absolute terms, the primary result is a reduction from 42% to 40% in the proportion of participants experiencing at least one acute respiratory tract infection. It seems unlikely that the general population would consider a 2% absolute risk reduction sufficient justification to take supplements. Furthermore, the definition of acute respiratory tract infection varied between studies, consisting of a mixture of diverse conditions such as acute otitis media, laboratory confirmed influenza, self-reported colds, parent reported colds or chest infections, or radiograph confirmed pneumonia.		

Khakshour et al.; 2015	Cross-sectional study	90 children below 5 years of age suffering from respiratory infections.		In the group of children with respiratory disorders, 9 (42.9%) exhibited vitamin D deficiency. Vitamin D deficiency showed no meaningful statistical relation with acute respiratory infections (*p* > 0.05).		It is assumed that the lack of significant differences in vitamin D is due to the gestational age and other factors except that vitamin D deficiency plays crucial roles in respiratory system infections.

Zittermann et al.; 2016	Review			Regarding acute respiratory tract infection, RCTs indicate a significant risk reduction by vitamin D supplements [OR = 0.65; 95% confidence interval (CI) 0.50–0.85]. There is evidence that daily administration is more effective than high-dose bolus administration [OR = 0.48 (95% CI 0.30–0.77) versus OR = 0.87 (95% CI 0.67–1.14)] and that individuals with deficient or insufficient (30–50 nmol/l) circulating 25-hydroxyvitamin D levels benefit most.	Innate and adaptive immunity	

**Table 3:** Studies on vitamin C, immunity, and common cold presented in the narrative review.

Authors	Type of study	Subjects	Dosage	Results	Type of immunity involved	Mechanism of action
Hemilä et al.; 2010	Review and meta-analysis	11306 men, woman and children	0.2 g daily for a single day or for a period	Twenty-nine comparisons examined the effect of prophylactic vitamin C on common cold duration (9649 episodes). In adults the duration of colds was reduced by 8% (3% to 12%), and in children by 13% (6% to 21%). The severity of colds was significantly reduced in the prophylaxis trials. Seven trial comparisons examined the effect of therapeutic vitamin C (3249 episodes). No consistent differences from the placebo group were seen in the duration or severity of colds.	All immunity	Regular ingestion of vitamin C had no effect on common cold incidence in the ordinary population. However, it had a modest but consistent effect in reducing the duration and severity of common cold symptoms. In trials with participants exposed to short periods of extreme physical stress (including marathon runners and skiers) vitamin C halved the common cold risk.

Maggini et al.; 2012	Double-blind, randomized, placebo-controlled pilot study	94 patients	1000 mg vitamin C plus 10 mg zinc	Rate of definite relief from rhinorrhoea was significantly higher in the active treatment group than in the placebo group over the 5-day assessment period (*p* = 0.03, Cox proportional hazard model).	All immunity	In view of the frequency of the common cold, coupled with the related social and economic costs and the limited treatment options, supplementation with vitamin C and zinc may represent an efficacious measure, with a good safety profile, to help ameliorate the symptoms of this infectious viral disease.

Penn et al.; 1991	Randomized controlled trial	Thirty elderly 30	100 mg for 28 days	*Percentage of cells (mean ± SD) in peripheral blood* *T cells*: 6.58 ± 6.3 (before) 69.1 ± 9.7 (after) *p* = NS *T4 cells*: 45.2 ± 4.5 (before) 50.3 ± 8.8 (after) *p* < 0.05 *T8 cells*: 24.7 ± 5.3 (before) 19.7 ± 7.6 (after) *p* < 0.05 *T4 : T8*: 1.92 ± 0.49 (before) 2.88 ± 1.2 (after) *p* < 0.01 *Proliferative response of lymphocytes to the mitogen PHA, at concentrations of 1/200 and 1/400, measured in decays per minute (dpm)* *PHA 1/200*: 9562 ± 11 (before) 15,690 ± 11,577 (after) *p* < 0.02 *PHA 1/400*: 3355 ± 3662 (before) 12,863 ± 11,872 (after) *p* < 0.01.	Innate immunity	Improvement in some aspects of cell-mediated immune function. In particular the number of T cells, T4 cells, and the T4 : T8 ratio increased significantly. The responsiveness of lymphocytes to the mitogen PHA also increased significantly and independently of the concentrations of the mitogen used. The results suggest that supplementation with physiological doses of vitamins A, C, and E in combination can improve cell-mediated immunity.

Schertling et al.; 1990	Randomized crossover trial	24 men and women	5 g/die for 35 days	The difference between the two groups (placebo period, ascorbic acid period) is statistically significant for the peak heights (*p* ~ 0.03). The changes in the alveolar macrophage activity measured on the basis of the formation of ROM do not correlate or only weakly correlate with the changes in peak flow values and symptom scores (|*r*| < 0.04 in all cases).	Innate immunity	In the presence of increased activity of the pulmonary inflammatory cells (e.g., alveolar macrophages, granulocytes) with bronchial asthma, the equilibrium between oxidative and antioxidative capacity in the lungs may be displaced in favor of the oxidative process, such that additional administration of ascorbic acid at a high dose (5 g/day) and over a longer period of time may be expected to provide a therapeutic effect.

Lauer et al.; 2013	placebo-controlled trial	33 healthy volunteers with skin types II and III according to the Fitzpatrick classification	100 or 180 mg for 4 weeks	*Carotenoid values before and after 4 weeks of vitamin C supplementation measured on the inner forearm and the palm* (Δ = 10^–4^ AU) Vitamin C dose 100 mg Total carotenoids Palm: 1.5 (*p* = NS) Forearm: 0.5 (*p* = NS) Lycopene Palm: 0.3 (*p* = NS) Forearm: −0.1 (*p* < 0.05) Vitamin C dose 180 mg Total carotenoids Palm: 0.6 (*p* = NS) Forearm: 0.8 (*p* < 0.05) Lycopene Palm: −0.1 (*p* < 0.05) Forearm: −0.1 (*p* = NS).	Skin	Vitamin C increases the antioxidative activity of the skin, and there is an increase in cutaneous carotenoids though it was not significant. The increase in cutaneous antioxidative activity occurred fast after supplementation and was enhanced with higher doses of vitamin C. The study shows that dietary supplementation with vitamin C can has a significant effect on skin radical scavenging.

Sasazuki et al.; 2006	A double-blind, 5-year randomized controlled trial	244 men and women	50 mg (low-dose group) or 500 mg (high-dose group)	When the common cold was defined as occurring three or more times during the survey period, an approximately 70% reduction in relative risks (RR) was observed (0.34, 95% CI: 0.12–0.97, *p* = 0.04). The corresponding value for the common cold defined as occurring four or more times was 0.28 (95% CI: 0.06–1.28, *p* = 0.10), for which only 10 events were observed. The results were essentially the same in the intention-to-treat groups.	All immunity	Vitamin C supplementation significantly reduces the frequency of the common cold but had no apparent effect on the duration or severity of the common cold.

Harper et al.; 2002	Case series study	12 men and women	1 g/day for 10 days	Reduction in spontaneous generation of superoxide (pretreatment 8.41 ± 0.7 nmol/10^6^ cells; posttreatment 5.64 ± 0.6 nmol/10^6^; *p* < 0.05). Total antioxidant capacity increased significantly following treatment with vitamin C (555.4 ± 142 versus 668.6 ± 186 *μ*mol/l trolox equivalent; *p* = 0.01) as did vitamin C concentration (56.5 ± 27 versus 137.7 ± 64 *μ*mol/l; *p* = 0.002).	Innate immunity	The treatment of patients with antioxidants reduced neutrophil generation of superoxide and suggested that antioxidants may have an important role as adjuvant therapy.

Nieman et al.; 2002	Randomized study	28 men and women	1500 mg/die for 7 days	Plasma ascorbic acid was markedly higher in the vitamin C compared with placebo group prerace and rose more strongly in the vitamin C group during the race (postrace: 3.21 ± 0.29 and 1.28 ± 0.12 *μ*g/100 *μ*l, resp., *p* < 0.001). No significant group or interaction effects were measured for lipid hydroperoxide, F2-isoprostane, immune cell counts, plasma interleukin (IL)-6, IL-10, IL-1-receptor antagonist, or IL-8 concentrations, or mitogen-stimulated lymphocyte proliferation and IL-2 and IFN-*γ* production.	Innate immunity	Vitamin C supplementation does not serve as a countermeasure to postrace oxidative and immune changes in carbohydrate fed ultramarathon runners. Statistical correlations suggest that oxidative stress had little influence on the immune changes that take place during or after a competitive ultramarathon race.

Davison and Gleeson.; 2006	Single blind, randomized crossover design	9 men	1000 mg for 2 weeks	Main effects *p* values (trial; time; interaction) ACTH (adrenocorticotrophic hormone) (pg ml^−1^) 0.303; 0.002; 0.276 IL-6 (pg ml^−1^) 0.818; 0.000; 0.795 Cortisol (nM) 0.097; 0.004; 0.039. There was a significant trial-time interaction effect for plasma cortisol concentration (*p* = 0.039). There was a significantly lower postexercise neutrophilia (*p* < 0.014) in the VC trial, compared with the PLA trial.	Innate immunity	Vitamin C (VC) was effective at increasing antioxidant defence, modulating the leukocytosis and neutrophilia responses and possibly had some small effects on the plasma cortisol response. This suggests that supplementation with VC alone for a period of up to 2 weeks provides very limited or no protection against the depression of neutrophil function which is typically observed after prolonged exercise.

Du et al.; 2003	Randomized controlled study	84 men and women	Treatment group: vit C (10 g/day) was given intravenously for 5 days control group: vit C (1 g/day) was given intravenously for 5 days	The ratios of CD_4_/CD_8_ and CD_4_ positive cells were decreased, especially in severe acute pancreatitis (SAP) patients (*p* < 0.05. CD_4_/CD_8_, *p* = 0.041; CD_4_, *p* = 0.019). After treatment, the average value of concentration of plasma vitamin C (P-VC) was significantly higher and the values of SIL-2R, TNF-*α*, IL-6, and IL-8 were significantly lower in the treatment group than in the control group (*p* < 0.05 P-VC, *p* = 0.045; SIL-2R, *p* = 0.012; TNF-*α*, *p* = 0.030; IL-6, *p* = 0.015; and IL-8, *p* = 0.043).	Innate immunity	High-dose vitamin C has therapeutic efficacy on acute pancreatitis. Compared with the normal group, CD_3_ and CD_4_ positive cells in acute pancreatitis (AP) patients were significantly decreased. The potential mechanisms include promotion of antioxidizing ability of AP patients, blocking of lipid peroxidation in the plasma, and improvement of cellular immune function.

Dunstan et al.; 2007	A randomized controlled trial	54 allergic adults	1500 mg/day for 4 weeks	Antioxidant supplementation resulted in significant increases in serum levels of vitamin C, vitamin E, *β*-carotene, and selenium levels, compared with the placebo group (*p* < 0.001). There was no change in serum antioxidative capacity (AC), plasma F2-isoprostanes, exhaled nitric oxide (eNO), or immune responses following supplementation with antioxidants compared with placebo.	Acquired immunity	Although the dietary supplement achieved changes in antioxidant levels, it did not result in any significant changes in established immune responses over the study period.

Hunter et al.; 2012	Randomized crossover study	32 older individuals	362.4 mg for 4 weeks	No changes to innate immune function (natural killer cell activity, phagocytosis) or inflammation markers (high-sensitivity C-reactive protein, homocysteine) were detected. *p* = 0.03 (significant value was defined a priori at *p* < 0.001).	Innate immunity	Consumption of gold kiwifruit enhanced the concentrations of several dietary plasma analytes, which may contribute to reduced duration and severity of selected URTI (upper respiratory tract infections) symptoms, offering a novel tool for reducing the burden of URTI in older individuals.

Vojdani et al.; 2000	Randomized clinical trial	20 men and women	500, 1000, or 5000 mg for 2 weeks	*Intracellular levels of ascorbic acid in human leukocytes* (*μ*g/5 × 10^6^ cells) *Dosage 500 mg*: (mean ± SD) Day 0: 6.8 ± 2.1 Day 1: 9.1 ± 1.1 *p* = 0.03 (*p* from day 0 to day 1) Day 7: 10 ± 1.4 *p* = 0.01 (*p* from day 1 to day 7) Day 15: 10.1 ± 1.2 *p* = 0.01 (*p* from day 7 to day 15) Day 21: 7.2 ± 1.7 *p* = 0.18 (*p* from day 15 to day 21) *Dosage 1000 mg*: (mean ± SD) Day 0: 7.4 ± 2.4 Day 1: 10 ± 2.6 *p* = 0.01 (*p* from day 0 to day 1) Day 7: 10.3 ± 2.7 *p* = 0.01 (*p* from day 1 to day 7) Day 15: 10.7 ± 2.8 *p* = 0.01 (*p* from day 7 to day 15) Day 21: 7.3 ± 2.3 *p* = 0.26 (*p* from day 15 to day 21) *Dosage 5000 mg*: (mean ± SD) Day 0: 7 ± 2.3 Day 1: 9.1 ± 1.8 *p* = 0.01 (*p* from day 0 to day 1) Day 7: 9.2 ± 1.7 *p* = 0.03 (*p* from day 1 to day 7) Day 15: 9.5 ± 1.9 *p* = 0.01 (*p* from day 7 to day 15) Day 21: 7 ± 2.6 *p* = 0.91 (*p* from day 15 to day 21) *NK cell cytotoxic activity (LU) * *Dosage 500 mg*: (mean ± SD) Day 0: 35.6 ± 27.6 Day 1: 55.8 ± 18.8 *p* = 0.07 (*p* from day 0 to day 1) Day 7: 50.6 ± 12.8 *p* = 0.19 (*p* from day 1 to day 7) Day 15: 52.4 ± 24.4 *p* = 0.05 (*p* from day 7 to day 15) Day 21: 39.6 ± 26.4 *p* = 0.01 (*p* from day 15 to day 21) *Dosage 1000 mg*: (mean ± SD) Day 0: 34.2 ± 22.5 Day 1: 72.6 ± 39.12 *p* = 0.04 (*p* from day 0 to day 1) Day 7: 82 ± 46.4 *p* = 0.04 (*p* from day 1 to day 7) Day 15: 77.6 ± 36.4 *p* = 0.04 (*p* from day 7 to day 15) Day 21: 43.2 ± 29.6 *p* = 0.17 (*p* from day 15 to day 21) *Dosage 5000 mg*: (mean ± SD) Day 0: 33.4 ± 28.8 Day 1: 67.4 ± 39 *p* = 0.19 (*p* from day 0 to day 1) Day 7: 56.6 ± 29.5 *p* = 0.21 (*p* from day 1 to day 7) Day 15: 58.8 ± 26.2 *p* = 0.24 (*p* from day 7 to day 15) Day 21: 35 ± 24.9 *p* = 0.60 (*p* from day 15 to day 21).	Innate immunity	We concluded that ascorbic acid in an antioxidant and doses up to 5000 mg neither induce mutagenic lesion nor have negative effects on NK cell activity.

McComsey et al.; 2003	Pilot trials	8 men and women	1000 mg/die for 24 weeks	*CD*_4_^+^ * cell count (cell/mm* ^*3*^) (mean ± SD) Study entry: 627 ± 316 Week 24: 605 ± 460 (*p* = 0.49).	Innate immunity	There is a rationale for testing of antioxidant but there is no statistical significance.
